# Serum endostatin is selectively reduced in non-steatotic chronic hepatitis C and normalizes after direct-acting antiviral therapy: a prospective cohort study

**DOI:** 10.1186/s12876-026-05500-2

**Published:** 2026-11-06

**Authors:** Kilian Weigand, Georg Peschel, Martina Müller, Christa Buechler

**Affiliations:** 1 Department of Internal Medicine I, Gastroenterology, Hepatology, Endocrinology, Rheumatology, and Infectious Diseases, University Hospital Regensburg, 93053 Regensburg, Germany; 2 Department of Gastroenterology, Gemeinschaftsklinikum Mittelrhein, 56073 Koblenz, Germany; 3 Department of Internal Medicine, Klinikum Fürstenfeldbruck, 82256 Fürstenfeldbruck, Germany

**Keywords:** Direct-acting antivirals, Angiogenesis, Steatotic liver disease, HCV

## Abstract

**Background:**

Endostatin is an endogenous inhibitor of angiogenesis that has been linked to chronic liver disease and proposed as a circulating marker of fibrosis in hepatitis C virus (HCV) infection. Although direct-acting antivirals (DAAs) reliably achieve viral eradication and improve liver-related outcomes, their effects on circulating endostatin have remained unknown.

**Materials and methods:**

Serum endostatin levels were measured in 92 patients with chronic HCV infection and compared with those in 30 healthy controls. In a longitudinal subgroup of 58 patients, samples were available before treatment, at weeks 4 and 12 of therapy, and at sustained virological response 12 (SVR12). Serum from 85 patients was analyzed at SVR12.

**Results:**

Patients with chronic HCV had significantly lower serum endostatin levels than healthy controls before therapy. This decrease was confined to patients without hepatic steatosis, whereas patients with steatotic liver disease exhibited endostatin levels comparable to those of healthy controls. Endostatin levels were not associated with advanced fibrosis or cirrhosis, as assessed by noninvasive fibrosis scores and ultrasound. In cirrhosis, serum endostatin levels were reduced after excluding patients with a glomerular filtration rate < 90 mL/min who had higher serum endostatin levels. During DAA therapy, serum endostatin increased in patients without steatosis and, by SVR12, reached levels similar to those in patients with steatotic liver disease and healthy controls. Endostatin was positively associated with age. After age adjustment, endostatin positively correlated with albumin, leukocyte and platelet counts, and negatively correlated with the model for end-stage liver disease score, bilirubin, international normalized ratio, glomerular filtration rate, and high-density lipoprotein. Because these variables did not differ between patients with and without steatotic liver disease, the differential regulation of endostatin in chronic HCV is likely driven by additional disease-specific mechanisms.

**Conclusions:**

This study showed that chronic HCV is associated with reduced serum endostatin levels in patients without steatosis, which normalize after successful DAA therapy. Serum endostatin is not a marker of fibrosis severity in chronic HCV, either before or after viral clearance. Serum endostatin levels are increased in patients with impaired renal function, which is a confounding factor in patients with liver disease. Together, these findings provide preliminary evidence for a previously unrecognized pattern of endostatin dysregulation in chronic HCV that is almost reversed after antiviral cure.

**Supplementary Information:**

The online version contains supplementary material available at https://doi.org/10.1186/s12876-026-05500-2.

## Introduction

Chronic hepatitis C virus (HCV) induces sustained hepatic inflammation, driving the onset and progression of liver fibrosis and hepatocellular carcinoma (HCC) [1, 2]. Angiogenesis is closely linked to fibrosis progression, and animal studies show that inhibiting angiogenesis protects against fibrosis development and portal hypertension [3]. Endostatin is a C-terminal peptide of collagen XVIII and a potent anti-angiogenic molecule highly abundant in hepatocytes [4, 5]. Endostatin binds to the vascular endothelial growth factor (VEGF) receptors 1 and 2, inhibiting VEGF binding and angiogenesis [4, 6]. Endostatin induces apoptosis in endothelial cells and inhibits endothelial cell proliferation and migration [4, 7]. Further studies have shown that endostatin directly affects hepatic stellate cells, the principal cells that produce the extracellular matrix [8–10]. Endostatin prevented activation of these cells, which is characterized by high expression of extracellular matrix proteins, upon stimulation with transforming growth factor beta or VEGF [9, 10].

However, data on the association between serum endostatin levels and liver fibrosis are inconsistent: levels increase with fibrosis progression, decline in patients with cirrhosis, and remain unchanged in patients with cirrhosis compared with healthy controls [11–16]. Liver cirrhosis did not influence endostatin levels in critically ill patients [17]. In patients with HCV, serum endostatin levels were higher than in healthy controls [18], and those with severe fibrosis had higher serum endostatin compared to those with mild fibrosis [19]. Although findings on fibrosis were inconsistent, most studies reported higher circulating endostatin levels in patients with HCC [13, 14, 16, 18].

Estimated glomerular filtration rate (GFR), a marker of renal function, decreases in patients with chronic hepatitis C virus (HCV) infection and is associated with liver disease severity [20, 21]. In addition, impaired kidney function has consistently been linked to elevated circulating endostatin levels [22]. Therefore, renal impairment may partly confound the observed relationship between endostatin concentrations and liver disease severity. Of note, in critically ill patients requiring dialysis, cirrhosis attenuated acute kidney injury-associated upregulation of plasma endostatin [17].

A study indicated that the spleen plays a regulatory role in circulating endostatin levels. It showed that splenic activity influences hepatic cathepsin S, which promotes cleavage of collagen XVIII, thereby enhancing endostatin production in fibrotic liver tissue [23]. Splenectomy in mice led to lower circulating concentrations of both cathepsin S and endostatin [23]. Similarly, in patients with liver cirrhosis, analysis of matched serum samples collected before and after splenectomy revealed a decline in cathepsin S and endostatin levels within one month following the procedure [23].

Clinical practice uses shear-wave elastography (SWE), which measures liver stiffness, and the fibrosis-4 (FIB-4) score, calculated from routine clinical measurements, to noninvasively assess liver fibrosis stages; both have been shown to be appropriate for diagnosing advanced fibrosis in patients with HCV [24, 25]. Nevertheless, the FIB-4 score may yield many inconclusive results and may underestimate or misclassify the extent of fibrosis, particularly in individuals with obesity. Moreover, these scores are also associated with inflammation and subsequently improve shortly after a successful viral cure [26, 27]. Combining the FIB-4 score and SWE offers a sequential, dual-assessment strategy that improves the accuracy of non-invasive liver fibrosis staging by compensating for each method’s limitations when used independently [28]. The diagnosis of cirrhosis should be based on an integrated assessment of underlying disease etiology, imaging findings, laboratory parameters, and non-invasive diagnostic tests. Although imaging-based morphological features have high specificity for cirrhosis, their sensitivity remains limited, as with other diagnostic criteria [29].

Direct-acting antivirals (DAAs) have revolutionized HCV management, achieving viral clearance rates exceeding 90% and high sustained virologic response (SVR) rates, even among patients with advanced liver fibrosis [30, 31]. In the long term, successful DAA therapy reduces the risk of fibrosis progression, HCC development, and overall mortality [32]. Whether serum endostatin levels also change during HCV eradication has not been tested.

The main aims of this study were to determine whether serum endostatin levels in patients with chronic HCV change during DAA treatment and whether these levels are associated with liver fibrosis/cirrhosis.

## Materials and methods

### Study cohort

This prospective cohort study enrolled patients with chronic HCV infection between October, 2014 and September 2017 at the Department of Internal Medicine I, University Hospital Regensburg. Eligible participants were adults (≥ 18 years) who were scheduled to receive direct-acting antiviral (DAA) therapy. All included patients were treatment-naïve with respect to HCV. Exclusion criteria comprised decompensated cirrhosis, as well as co-infection with the hepatitis B virus or the human immunodeficiency virus.

**Fig. 1:**
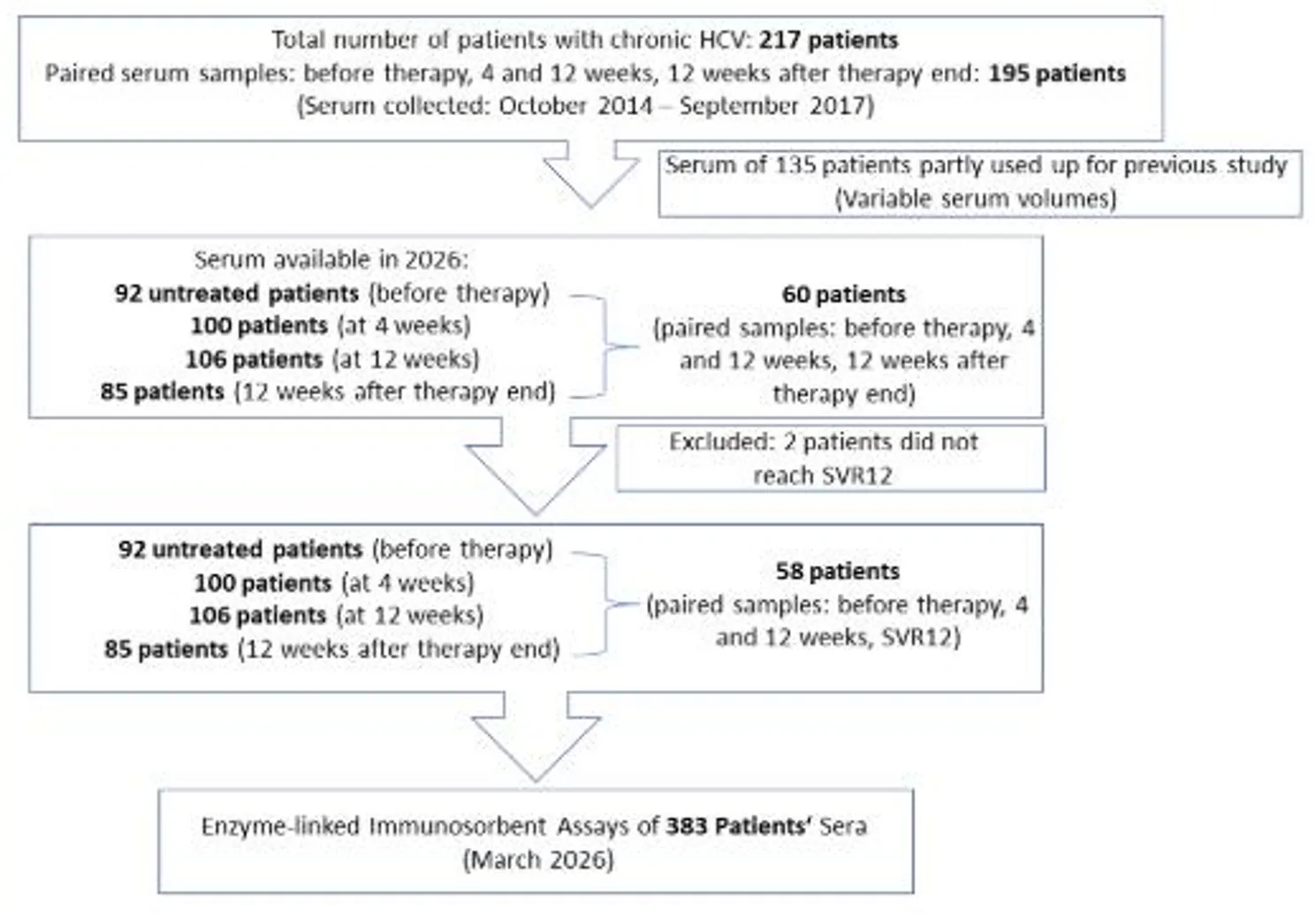
Study cohort (HCV, hepatitis C virus; SVR12, sustained virological response 12)

Serum samples were available for 195 of the initially enrolled 217 patients before therapy started, and at 4 and 12 weeks during therapy, as well as at SVR12 (Fig. 1). These serum samples were used in previous studies to measure various substances, including serum lipids, gremlin 1, omentin-1, galectin-3, soluble CD137, proprotein convertase subtilisin/kexin 9, chemerin, tumor necrosis factor, interleukin-4, interleukin-10, interferon-gamma-inducible protein 10, and interferon alpha. These studies cannot be cited here to avoid high self-citation. Because serum volumes varied across time points, most samples were unavailable for the current study. For analysis of endostatin, serum samples were available from 92 treatment-naïve patients at baseline, 100 patients after 4 weeks of therapy, and 106 patients at the end of treatment (12 weeks). In addition, serum samples collected 12 weeks after completion of therapy were available for 85 patients (Fig. 1). A control group of 30 healthy individuals with a median age of 56 years (range 21–67), comparable to the HCV cohort (*p* = 0.450) was also analyzed. The control group included 19 females and 11 males, with no significant difference in sex distribution compared with the patient population (*p* = 0.102).

### Ultrasound examination and calculation of the fibrosis-4 score

The fibrosis-4 (FIB-4) index was applied as a validated non-invasive marker of hepatic fibrosis. According to established thresholds, a FIB-4 value > 3.25 indicates fibrosis, whereas values < 1.3 in individuals younger than 65 years and < 2.0 in those older than 65 years exclude significant fibrosis [33]. Liver stiffness was additionally assessed using dynamic shear-wave elastography (SWE). Measurements were performed with the GE LOGIQ E9 (1–6 MHz probe), Siemens Acuson S2000 (1–5 MHz probe), and Siemens Acuson S3000 (1–6 MHz probe) systems. For each patient, 8 to 12 valid readings were obtained, and the median value was used for analysis. Fibrosis stages were assigned based on established cut-off values described by Colombo et al. [34].

Distinguishing cirrhosis from high-grade liver fibrosis can be challenging. This diagnostic continuum is reflected in the concept of “advanced chronic liver disease,” introduced in the Baveno VI consensus, which acknowledges the transition between advanced fibrosis and cirrhosis [35]. The FIB-4 score, a commonly used non-invasive test, has a high negative predictive value for cirrhosis (96%) but only a limited positive predictive value (63%), as documented by Parikh et al. [36]. Another important non-invasive test is liver stiffness measurement by ultrasound-based elastography. Most pooled studies evaluating elastography have used vibration-controlled transient elastography (VCTE) and report liver stiffness measurements > 15 kPa as a cutoff with high specificity (95%) for cirrhosis [37]. In the present study, however, two-dimensional shear-wave elastography (2D-SWE) was used. Because cutoff values for 2D-SWE may vary by manufacturer, we applied the pooled cutoff values reported by Colombo et al. [34], which were available at the time of patient inclusion and consistent with the manufacturer’s recommendations. More recent studies have begun to define more harmonized cutoff values across ultrasound systems and SWE platforms from different manufacturers [38]. Accordingly, cirrhosis diagnosis should rely on a combination of parameters, including imaging findings, laboratory results, disease etiology, and non-invasive tests. Imaging-based morphological criteria remain highly specific, although, like other criteria, they show limited sensitivity for diagnosing cirrhosis [29]. Liver biopsy was reserved for unclear cases because of its invasive nature and the availability of non-invasive alternatives, as discussed above, and was therefore not included systematically for ethical reasons. Consequently, we chose a multifactorial approach to define cirrhosis, based on a combination of sonographic features and laboratory findings documented in the patients’ medical records by our Department of Hepatology.

Steatosis was defined as a hyperechogenic liver with a bright hepatic echo pattern compared with the right kidney, with or without attenuation of the ultrasound beam and with or without impaired visibility of the diaphragm (diaphragmatic attenuation) and the portal vein tract (vascular blurring). The severity of steatosis was not visually quantified, for example, using the semi-quantitative approach proposed by Ibacahe et al. [39] or ultrasound-based quantitative methods such as ultrasound-guided fat quantification (UGAP)^®^, because UGAP was not yet available at the study center at the time of the study.

The study was conducted from 2014 to 2017; during this time, the GE-driven system for UGAP was not evaluated for clinical use, and the corresponding white paper was published some years later [39]. Furthermore, our institution did not have the controlled attenuation parameter (CAP). Therefore, we defined steatosis based on the visual appearance of a bright liver compared with the kidney texture. Indeed, a bright liver echo pattern shows a low sensitivity of 64% but a high specificity of 97% for liver steatosis. However, sensitivity increases to 91% for detecting high-grade steatosis of > 30%, according to Palmentieri et al. [40]. Well-validated semiquantitative scoring systems, such as those proposed by Ibacahe et al. [39] to grade steatosis (for example, by brightness or visibility of the diaphragm or liver vessels), were not available when the study was performed and cannot be applied retrospectively.

The presence of metabolic dysfunction–associated steatotic liver disease (MASLD) in the patient cohort was not documented, since data on the diagnosis of non-alcoholic fatty liver disease, as captured during patient enrollment, were not collected in this study.

### Endostatin ELISA

Serum aliquots were stored at -80 °C and were thawed immediately prior to use. Serum was diluted 1:100 for endostatin measurement with the human endostatin DuoSet ELISA Kit (Catalog #: DY1098; Biotechne, Wiesbaden, Germany). Standards and serum samples were all measured in duplicate and the mean values were used for calculations.

We used this ELISA to measure serum and plasma endostatin levels of 18 controls. The median plasma concentration was 122 ng/ml and the median serum concentration was 126 ng/ml; these values did not differ significantly (*p* > 0.05). Comparisons of studies using either serum or plasma did not reveal consistent differences between serum and plasma endostatin levels [41].

### Statistics

Serum endostatin levels were not normally distributed according to the Shapiro- Wilk test (*p* = 0.031). Results are displayed as boxplots summarizing the minimum, maximum, median, and interquartile range (first and third quartiles). Outliers are shown as individual points or an asterisk beyond the whiskers. Tabulated data are reported as medians with corresponding minimum and maximum values in parentheses. The interquartile range is included where appropriate. For statistical comparisons, the nonparametric Mann–Whitney U test and Kruskal–Wallis test (with post hoc Bonferroni correction) were applied to evaluate differences between two independent groups and among multiple independent groups, respectively (SPSS Statistics 31.0.0.0; IBM Corp., Armonk, NY, USA, 2025). Paired observations were analyzed using the Friedman test (SPSS Statistics 31.0.0.0). A Chi-squared test was used to compare categorical variables. Correlation analyses were performed using Spearman’s rank correlation coefficient (pairwise deletion of cases; SPSS Statistics 31.0.0.0). These values were corrected using the Benjamini-Hochberg correction. Linear regression was used to identify factors predicting serum endostatin levels. A p-value below 0.05 was considered statistically significant. As this study was exploratory, multiple-comparison correction was only performed for the correlation analysis and the Kruskal–Wallis test.

## Results

### Serum endostatin levels of patients with chronic HCV and controls

**Fig. 2:**
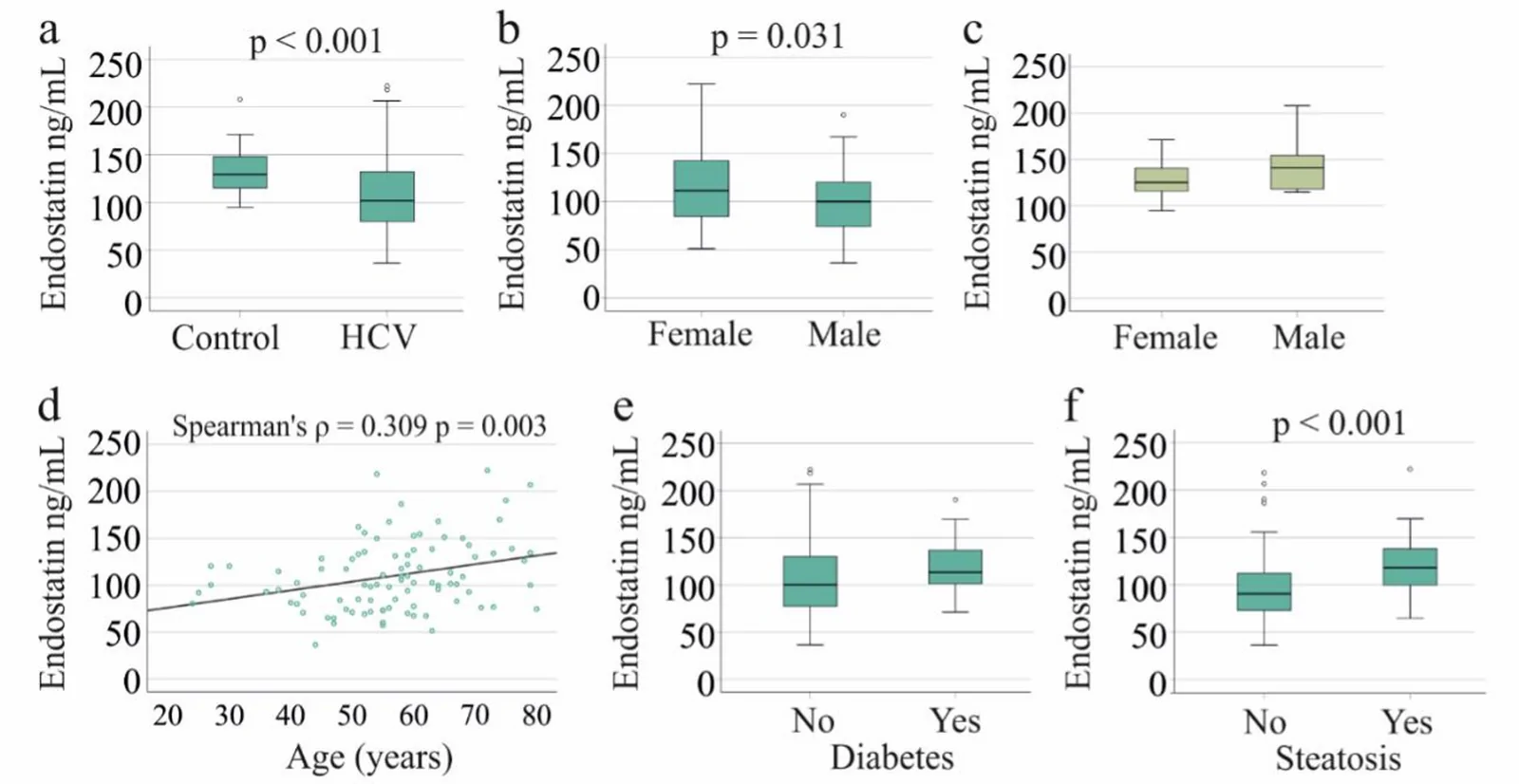
Serum endostatin levels of controls and patients with chronic hepatitis C virus (HCV) stratified for sex, type 2 diabetes and liver steatosis: (**a**) Serum endostatin levels of 30 controls and 92 HCV patients; (**b**) Serum endostatin levels of 50 male and 42 female HCV patients; (**c**) Serum endostatin levels of 11 male and 19 female controls; (**d**) Correlation of serum endostatin levels of 92 HCV patients with age; (**e**) Serum endostatin levels of HCV patients with (15 patients) and without (77 patients) type 2 diabetes; (**f**) Serum endostatin levels of HCV patients with (44 patients) and without (48 patients) steatosis. The small circles above the boxes mark outliers. Statistical test: Mann–Whitney U test and Spearman correlation in d

This study included 92 untreated patients with chronic HCV and 30 controls. Serum endostatin levels in the controls were 129 (95–208) ng/mL, higher than in patients, who had 102 (36–222) ng/mL (Figure 2a). 

In the patient cohort, serum endostatin levels were higher in 42 females than in 50 males (*p* = 0.031; Fig. 2b). In the control cohort, the 19 females and 11 males had similar serum endostatin levels (*p* = 0.254; Fig. 2c). In controls, serum endostatin did not correlate with age (*r* = -0.094, *p* = 0.619), whereas in patients, it was positively associated with age (*r* = 0.309, *p* = 0.003; Fig. 2d). Patients’ body mass index (BMI) did not correlate with endostatin levels (*r* = 0.060, *p* = 0.602).

### Serum endostatin levels in patients with HCV and liver steatosis or type 2 diabetes and correlation with spleen size

In the patient cohort, 15 patients had type 2 diabetes, but serum endostatin levels were similar to those of non-diabetic patients (*p* = 0.100; Fig. 2e). Patients with diabetes were older (*p* = 0.008). In patients older than 60 years, age (*p* = 0.787) and endostatin levels (*p* = 0.574) were similar between the 10 patients with diabetes and the 21 without.

The 44 patients with liver steatosis had higher serum endostatin levels than those without fatty liver (Fig. 2f). Serum endostatin was 90 (36–218) ng/mL in patients without liver steatosis and 118 (65–222) ng/mL in those with fatty liver (*p* < 0.001), and the levels in patients with steatosis were similar to those in healthy controls (129 (95–208) ng/mL, *p* = 0.050).

Patients with fatty liver had higher BMI and lower alanine aminotransferase levels, but did not otherwise differ from patients without liver steatosis (Table 1).

**Table 1:** Gender distribution, age, body mass index, model for end-stage liver disease (MELD) score, and laboratory parameters of patients with and without liver steatosis before therapy. If laboratory values were not obtained for all patients, the number (n) of patients for whom these measures were known is reported in superscript. Continuous data are presented as the median (minimum–maximum), with the interquartile range (IQR) also reported. Abbreviations: GFR, glomerular filtration rate; INR, international normalized ratio. Statistical Test: Mann-Whitney U test and chi-squared test for sex distribution

Parameter	Without Steatosis	With Steatosis	*p*-value
Gender (Female / Male)	19 / 29	23 / 21	0.156
Body mass index kg/m^2^	25.5 (18.4–34.6)^42^, IQR: 6.3	29.1 (18.4–40.4)^37^, IQR: 6.7	0.015
Age years	55 (24–80), IQR: 18	59 (27–78), IQR: 13	0.107
MELD Score	8 (6–19), IQR: 2	7 (6–13), IQR: 2	0.761
Alanine aminotransferase U/L	80 (27–287), IQR: 82	57 (22–247), IQR: 75	0.022
Aspartate aminotransferase U/L	75 (15–1230), IQR: 70	56 (14–238), IQR: 55	0.346
Bilirubin mg/dL	0.6 (0.2–4.3), IQR: 0.6	0.7 (0.3–2.9), IQR: 0.4	0.234
Albumin g/L	38.3 (19.9–45.4), IQR: 7.0	36.6 (19.0–45.5), IQR: 5.0	0.163
INR	1.1 (0.9–2.3), IQR: 0.2	1.1 (0.9–1.4), IQR: 0.2	0.656
Creatinine mg/dl	0.8 (0.1–1.1), IQR: 0.3	0.8 (0.5–1.3), IQR: 0.2	0.971
GFR mL/min	101 (66–161), IQR: 26	97 (47–131), IQR: 12	0.224
Leukocyte number/L	6.4 (2.2–10.5), IQR: 2.5	5.8 (2.6–12.3), IQR: 2.2	0.574
C-reactive protein mg/L	2.9 (2.9–7.8), IQR: 0.0	2.9 (2.9–29.9), IQR: 0.7	0.132
Procalcitonin ng/mL	0.07 (0.00–10.07), IQR: 0.11	0.08 (0.01–1.41), IQR: 0.10	0.293
Platelet number/nL	158 (38–302), IQR: 134	142 (43–364), IQR: 166	0.931
High-density lipoprotein mg/dL	51 (26–103) ^45^, IQR: 21	52 (19–82) ^39^, IQR: 26	0.993
Low-density lipoprotein mg/dL	91 (35–204) ^45^, IQR: 59	90 (31–179) ^39^, IQR: 39	0.964
Viral load	7 × 10^5^ (2600–11 × 10^6^), IQR:18 × 10^5^	6 × 10^5^ (23–25 × 10^6^), IQR: 17 × 10^5^	0.430

The prevalence of type 2 diabetes (*p* = 0.090) and liver steatosis (*p* = 0.156) did not differ between sexes.

Serum endostatin levels were not correlated with spleen size in 90 patients (*r* = -0.079, *p* = 0.460). This was also not the case when excluding patients with steatosis (*r* = -0.001, *p* = 0.996).

### Serum endostatin levels in patients with HCV in relation to glomerular filtration rate and platelet count

**Fig. 3:**
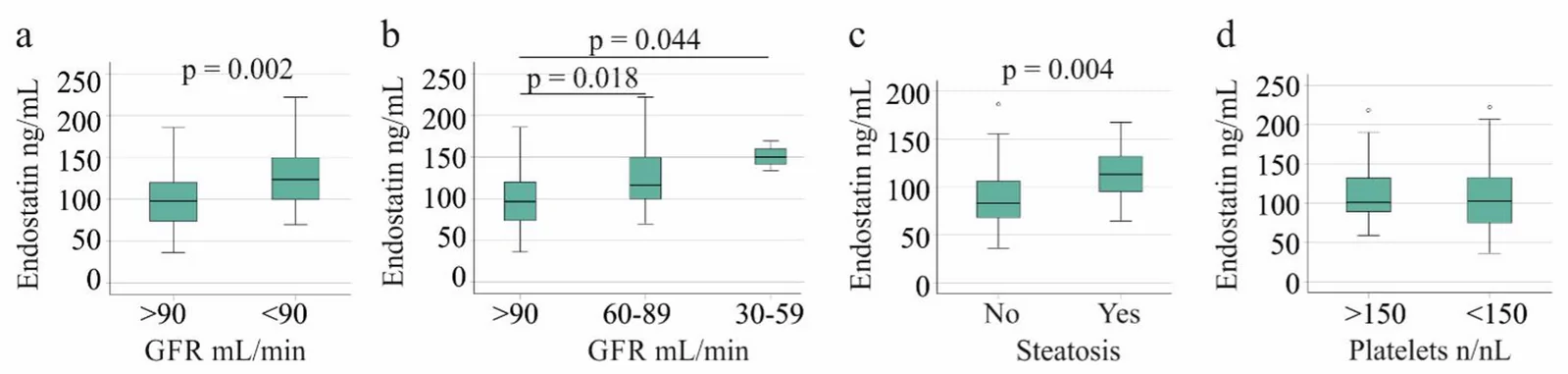
Serum endostatin levels of patients with chronic hepatitis C virus (HCV) in relation to renal function and platelet count: (**a**) Serum endostatin levels of 30 patients with GFR < 90 mL/min and 62 patients with GFR > 90 mL/min; (**b**) Serum endostatin levels of 62 patients with GFR > 90 mL/min, 27 patients with GFR between 89 and 60 mL/min and 3 patients with a GFR between 30 and 59 mL/min; (**c**) Serum endostatin levels of patients with GFR > 90 mL/min stratified for steatosis; (**d**) Serum endostatin levels of 47 patients with platelet count < 150/nL and 45 patients with platelet count > 150/nL; Statistical test: Mann–Whitney U test and Kruskal-Wallis test

Endostatin is increased in patients with renal disease [42] and was higher in the 30 patients with GFR < 90 mL/min than in the 62 with GFR > 90 mL/min (*p* = 0.002; Fig. 3a). Endostatin was higher in patients with a GFR between 60 and 89 mL/min (27 patients) and those with a GFR between 30 and 59 mL/min (3 patients) than in those with a GFR > 90 mL/min (62 patients; Fig. 3b). Excluding patients with GFR < 90 mL/min, the 29 patients with liver steatosis had higher serum endostatin than those without steatosis (113 (65–167) ng/mL versus 83 (36–186) ng/mL; *p* = 0.004; Fig. 3c).

However, the 47 patients with a low platelet count (< 150/nL) had serum endostatin levels similar to those of the 45 patients with levels > 150/nL (Fig. 3d, *p* = 0.397).

Because age strongly correlated with GFR (r = -0.722, p < 0.001) and INR with platelet count (r = - 0.746, p < 0.001), we did not include age or INR in the linear regression. In the entire HCV cohort, sex, BMI, platelet count, leukocytes, GFR, steatosis (Yes/No), and cirrhosis (Yes/No) predicted serum endostatin levels (F(7,70)=5.563, p < 0.001). GFR (p < 0.001) and steatosis (p = 0.011) were significant.In the non-steatotic HCV cohort, sex, BMI, platelet count, leukocytes, GFR, and cirrhosis (Yes/No) did not predict serum endostatin levels (p = 0.073). In the HCV cohort with steatosis, sex, BMI, platelet count, leukocytes, GFR, and cirrhosis (Yes/No) predicted serum endostatin levels (F(6,30) = 4.252, p = 0.003), with GFR (p = 0.003) and cirrhosis (p = 0.017) as significant factors.

### Serum endostatin levels in patients with HCV and liver fibrosis/cirrhosis

**Fig. 4:**
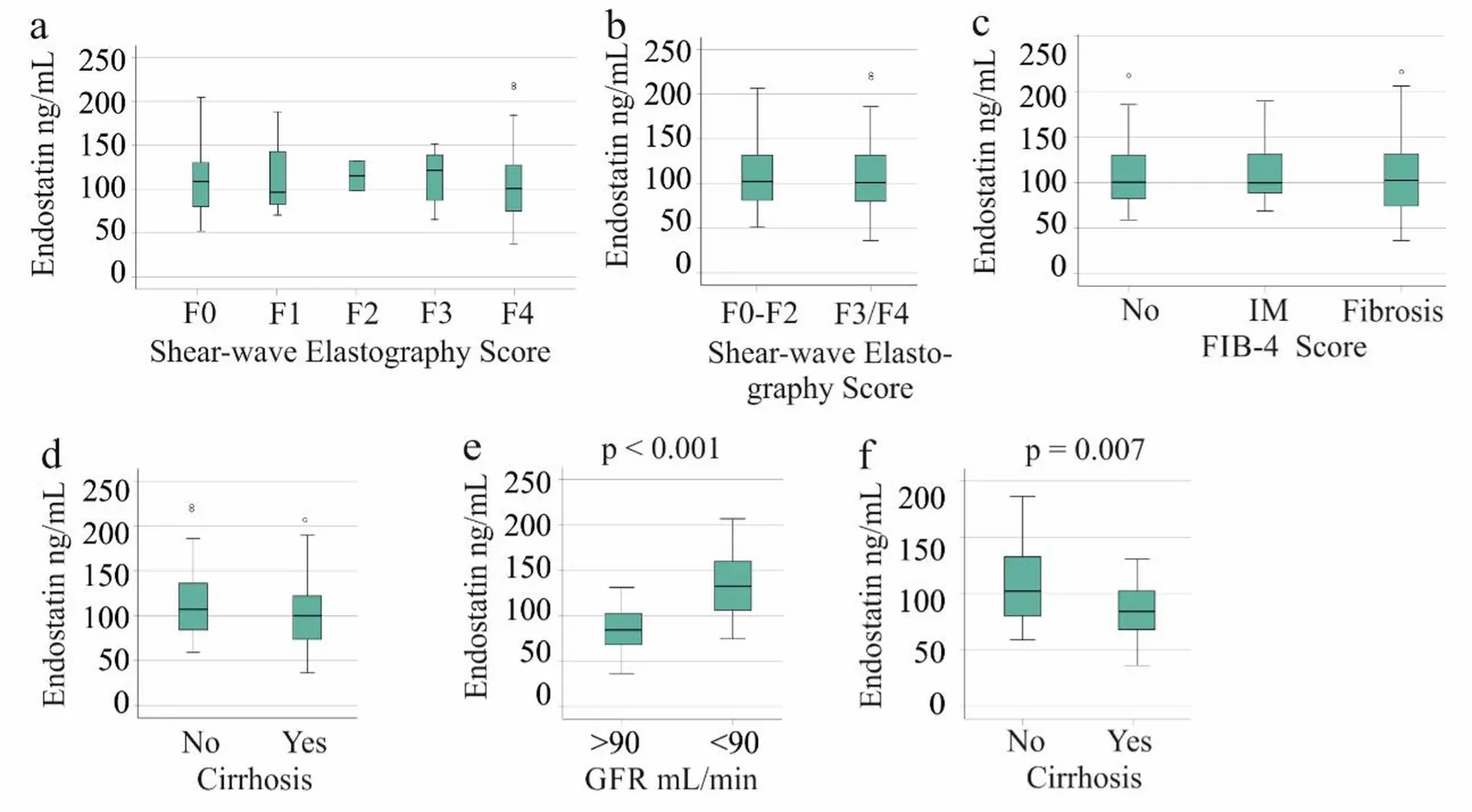
Serum endostatin levels in patients with chronic hepatitis C virus (HCV), stratified by fibrosis scores: (**a**) Serum endostatin levels of patients with increasing fibrosis stages defined by shear-wave elastography (29 patients with F0, 12 with F1, 2 with F2, 15 with F3, and 34 with F4); (**b**) Serum endostatin levels of patients with fibrosis stages F0 – F2 and F3/F4 defined by shear-wave elastography (43 patients with F0 – F2 and 49 with F3/F4); (**c**) Serum endostatin levels of patients with increasing fibrosis stages defined by the fibrosis-4 (FIB-4) score (28 patients were categorized as F0, 19 as intermediate (IM), and 45 as fibrosis); (**d**) Serum endostatin levels of patients with (36 patients) and without (56 patients) liver cirrhosis defined by ultrasound; (**e**) Serum endostatin levels of patients with cirrhosis who had GFR > 90 mL/min (24 patients) and who had GFR < 90 mL/min (12 patients); (**f**) Serum endostatin of patients with (24 patients) and without liver cirrhosis (38 patients) in the subgroup with GFR > 90 mL/min. The small circles above the boxes mark outliers. Statistical test: Kruskal–Wallis test in a and c, and Mann–Whitney U test in b, d, e and f

We assessed liver fibrosis using non-invasive approaches: shear-wave elastography (SWE) and the Fibrosis-4 (FIB-4) index. Based on SWE results, fibrosis stages were distributed as follows: 29 patients with F0, 12 with F1, 2 with F2, 15 with F3, and 34 with F4. Circulating endostatin concentrations were similar across groups (Fig. 4a). Ages (*p* = 0.294), GFR (*p* = 0.827), and platelet count (*p* = 0.027; no pairwise comparisons were significant after Bonferroni correction) were comparable. After excluding patients with liver steatosis or patients with GFR < 90 mL/min, endostatin levels were similar across fibrosis scores (*p* > 0.05 for both). Serum endostatin levels did not differ when patients with F0, F1, or F2 were combined and compared to patients with F3 or F4 fibrosis ) (Fig. 4b) (*p* = 0.922 for endostatin, *p* = 0.719 for age, *p* = 0.137 for platelet count, *p* = 0.650 for GFR).

Using the FIB-4 classification, 28 patients were categorized as F0, 19 as intermediate, and 45 as fibrosis. Serum endostatin levels did not vary significantly across these groups (Fig. 4c). This did not change after excluding patients with liver steatosis (*p* = 0.682). GFR did not change with the FIB-4 score (*p* = 0.170). However, platelet count decreased with higher FIB-4 scores (*p* < 0.001), and age increased with higher FIB-4 scores (*p* < 0.001) (age and platelet count used for the calculation of the FIB-4 score).

We also performed age-adjusted analyses. In patients younger than 60 years, 25 patients were categorized as F0, 14 as intermediate, and 17 as fibrosis. No significant variations in serum endostatin levels (*p* = 0.393), GFR (*p* = 0.696), or age (*p* = 0.140) were detected across these groups. Platelet count decreased with higher FIB-4 score (*p* < 0.001).

The subgroup of 36 patients with ultrasound-diagnosed liver cirrhosis had serum endostatin levels (100 (36–207) ng/mL) similar to those without cirrhosis (107 (59–222) ng/mL; Fig. 4d). GFR did not differ between patients with and without cirrhosis (*p* = 0.318). Excluding patients with liver steatosis, the 18 patients with cirrhosis and the 30 without had similar serum endostatin levels (*p* = 0.233).

In critical illness, the association between endostatin and renal dysfunction is attenuated in liver cirrhosis [17]. However, in HCV cirrhosis patients, those with lower GFR (12 patients) had much higher endostatin levels than those with higher GFR (24 patients, *p* < 0.001; Fig. 4e). In the subgroup of patients with GFR > 90 mL/min, the 24 patients with cirrhosis had lower serum endostatin than the 38 patients without cirrhosis (*p* = 0.007; Fig. 4f).

However, advanced fibrosis assessed by SWE or FIB-4 scores was not associated with lower serum endostatin levels in patients with GFR > 90 mL/min (*p* > 0.05 for both).

Patients with and without liver steatosis had similar prevalence of liver cirrhosis, SWE scores, and FIB-4 scores (*p* > 0.05 for all comparisons). Spleen size did not differ between patients with and without fatty liver (*p* = 0.289).

### Serum endostatin levels, viral load, and genotype

**Fig. 5:**
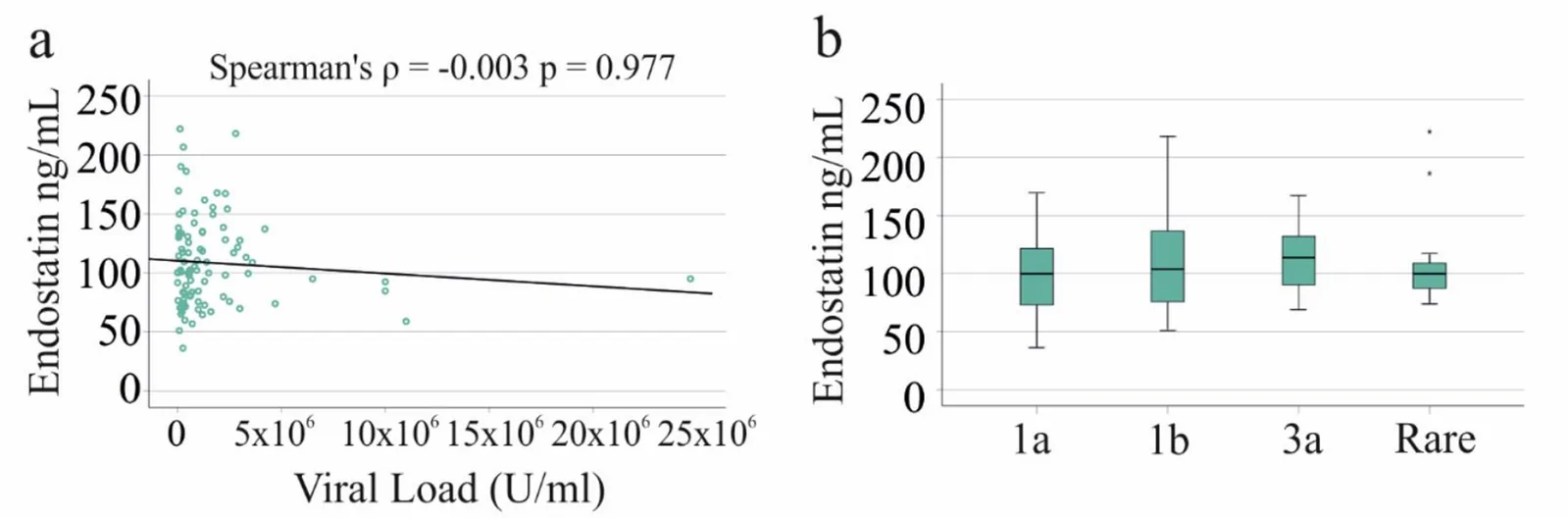
Serum endostatin levels in patients with chronic hepatitis C virus (HCV) in relation to viral load and stratified by viral genotype. **a** Correlation of serum endostatin with viral load in 92 untreated patients with HCV; (**b**) Serum endostatin levels in relation to viral genotype: 1a (25 patients), 1b (40 patients), 3a (16 patients), rare genotypes (11 patients). Small asterisks above the boxes mark outliers. Statistical test: Spearman correlation and Kruskal–Wallis test

Serum endostatin levels were not correlated with viral load (*r* = -0.003, *p* = 0.977, Fig. 5a). The HCV genotypes included 1a (25 patients), 1b (40 patients), and 3a (16 patients), while rare genotypes were grouped together (11 patients). Serum endostatin levels did not differ among HCV genotypes across the entire cohort (*p* = 0.741; Fig. 5b), among patients without steatosis (*p* = 0.576), among patients with steatosis (*p* = 0.546), or among patients with GFR > 90 mL/min (*p* = 0.289).

### Serum endostatin levels and measures of inflammation and liver function

**Fig. 6:**
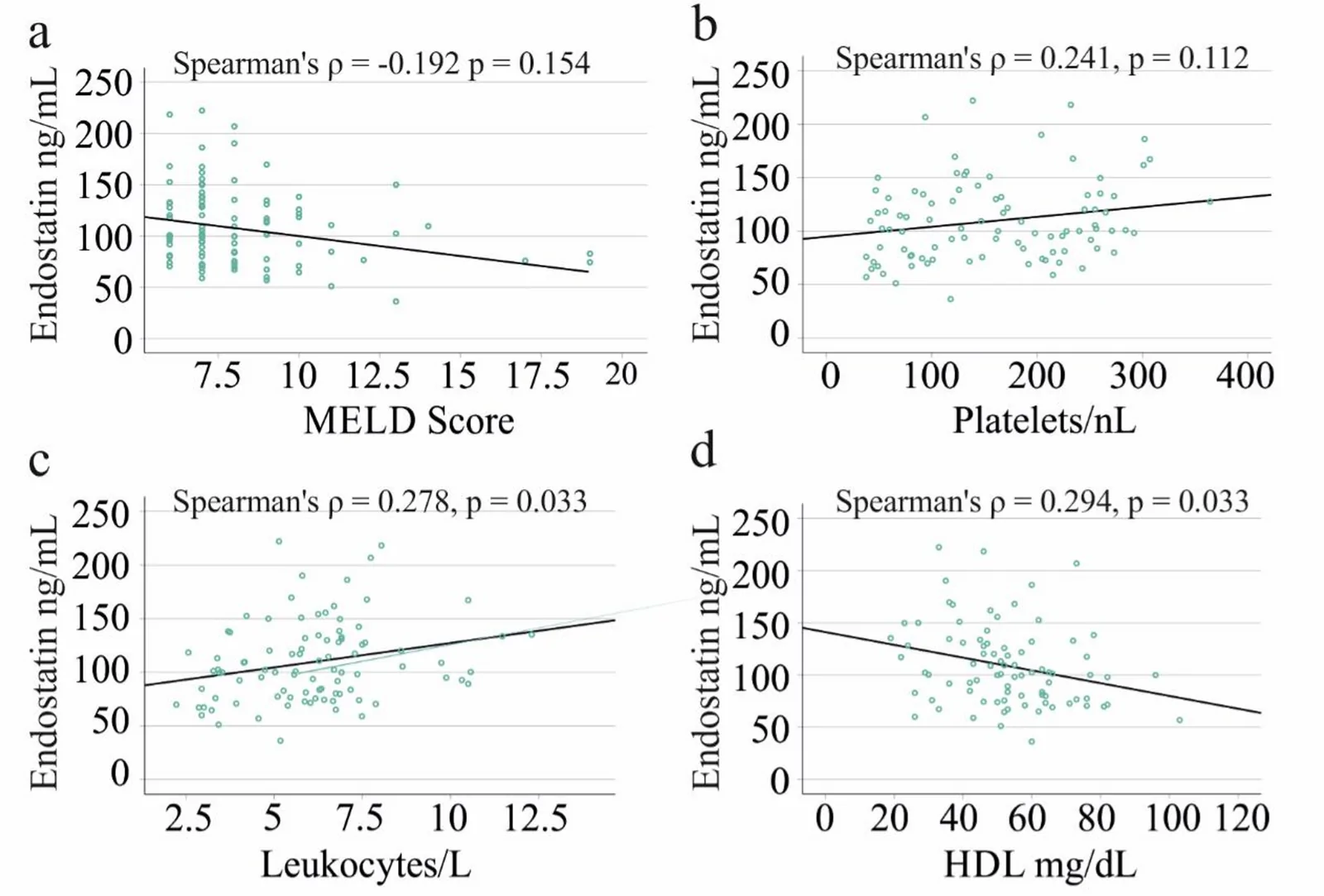
Correlation of serum endostatin with the model for end-stage liver disease (MELD) score, platelet and leukocyte counts, and high-density lipoprotein (HDL) in patients with hepatitis C virus (HCV). **a** Correlation of serum endostatin with the MELD score in 92 untreated patients with HCV; (**b**) Correlation of serum endostatin with platelet count in 92 untreated patients with HCV; (**c**) Correlation of serum endostatin with leukocyte number in 92 untreated patients with HCV; (**d**) Correlation of serum endostatin with HDL in 84 untreated patients with HCV. Statistical test: Spearman correlation. The p-values were Benjamini-Hochberg corrected

In the patient cohort, serum endostatin levels did not correlate with the model for end-stage liver disease (MELD) score (Fig. 6a), bilirubin, albumin, AST, or ALT, or international normalized ratio (INR) (Table 2). It did not correlate with platelet count (Fig. 6b) and positively correlated with leukocytes (Fig. 6c). Serum endostatin levels were weakly negatively correlated with the glomerular filtration rate (GFR) and high-density lipoprotein (HDL; Fig. 6d) (Table 2).

**Table 2:** Spearman correlation coefficients and corresponding p-values for the associations between serum endostatin levels and laboratory measures in 92 untreated patients with chronic hepatitis C. Correlation analyses were also performed after adjustment for age in the entire cohort, as well as separately in patients with and without hepatic steatosis. Superscript numbers indicate laboratory parameters that were not available for all patients and denote the number of patients for whom the respective measurement was recorded. Abbreviations: ALT, Alanine aminotransferase; AST, Aspartate aminotransferase; CRP, C-reactive protein; GFR, glomerular filtration rate; HDL, High-density lipoprotein; INR, International normalized ratio; LDL, Low-density lipoprotein; MELD, Model for End-Stage Liver Disease. All p-values were Benjamini-Hochberg corrected

Parameter	Correlation coefficient	*p*-value	Correlation coefficient	*p*-value	Correlation coefficient	*p*-value	Correlation coefficient	*p*-value
	Entire Cohort (92 patients)		Entire Cohort (92 patients)		Without Steatosis (48 patients)		With Steatosis (44 patients)	
	Unadjusted		Adjusted for Age		Adjusted for Age			
MELD Score	-0.192	0.154	-0.392	0.004	-0.392	0.032	-0.340	0.089
ALT U/L	-0.136	0.306	-0.073	0.575	-0.082	0.627	-0.008	0.962
AST U/L	-0.023	0.826	0.063	0.620	0.090	0.627	0.094	0.640
Bilirubin mg/dL	-0.048	0.700	-0.279	0.016	-0.246	0.222	-0.307	0.089
Albumin g/L	0.146	0.292	0.339	0.004	0.405	0.032	0.266	0.148
INR	-0.227	0.105	-0.402	0.004	-0.397	0.032	-0.317	0.089
Creatinine mg/dL	0.103	0.420	0.127	0.326	0.113	0.627	0.148	0.480
GFR ml/min	-0.313	0.033	-0.264	0.026	-0.221	0.325	-0.271	0.148
Leukocytes/L	0.278	0.033	0.321	0.006	0.386	0.032	0.258	0.148
CRP mg/L	0.109	0.419	0.012	0.913	0.199	0.325	-0.104	0.644
Procalcitonin ng/mL	0.089	0.470	-0.082	0.565	-0.068	0.653	0.001	0.962
Platelets/nL	0.241	0.112	0.352	0.004	0.320	0.078	0.396	0.061
HDL mg/dL	-0.294^74^	0.033	-0.249^74^	0.039	-0.101^45^	0.627	-0.468^39^	0.042
LDL mg/dL	0.177^74^	0.216	0.245^74^	0.326	0.193^45^	0.325	0.337^39^	0.089

Partial correlation, adjusted for age, which was positively correlated with serum endostatin levels (Fig. 2d), showed that endostatin remained significantly correlated with leukocytes, GFR, and HDL (Table 2). In the age-adjusted analysis, endostatin was negatively correlated with the MELD score, bilirubin, and INR, and positively correlated with albumin and platelet count (Table 2).

Negative correlations of endostatin with the MELD score and INR, and positive correlations with albumin, leukocytes, were significant in patients without liver steatosis (Table 2). In patients with liver steatosis, the negative correlation with HDL was significant (Table 2).

### Association of serum endostatin levels with viral cure

Paired serum samples from 60 patients were available at the start of therapy, at 4 and 12 weeks during DAA treatment, and at 12 weeks after therapy ended (Fig. 1). Direct-acting antiviral (DAA) therapy effectively eliminated HCV in 58 patients, resulting in a significant reduction in viral load at SVR12 [43] (Table 3). ALT, AST, and procalcitonin were lower, whereas albumin and LDL were higher at SVR12 than at pretreatment levels (Table 3).

**Table 3:** Gender distribution, age, body mass index, model for end-stage liver disease (MELD) score, and laboratory parameters of 58 patients with serum available before and during DAA therapy, as well as at SVR12. If laboratory values were not obtained for all patients, the number (n) of patients for whom these measures were known is reported in uppercase. Continuous data are given as the median (minimum value –maximum) and the interquartile range (IQR). Abbreviations: GFR, glomerular filtration rate; INR, international normalized ratio; SVR12, sustained virological response 12. Statistical test: Friedman test

Parameter	Before therapy (58 patients)	SVR12 (58 patients)	*p*-value
Gender (F / M)	28/30	28/30	> 0.05
Body mass index kg/m^2^	25.7 (18.4–40.4)^48^, IQR: 6.7	25.7 (18.4–40.4)^48^, IQR: 7.1	> 0.05
Age years	57 (27–79), IQR: 13	57 (27–79), IQR: 12	> 0.05
MELD Score	7 (6–13), IQR: 2	7 (6–13), IQR: 2	> 0.05
Alanine aminotransferase U/L	72 (22–240), IQR: 82	23 (6–135), IQR: 13	< 0.001
Aspartate aminotransferase U/l	53 (14–208), IQR: 51	21 (10–76), IQR: 12	< 0.001
Bilirubin mg/dL	0.6 (0.3–2.0), IQR: 0.4	0.6 (0–2.8), IQR: 0.6	> 0.05
Albumin g/L	36.8 (28.3–45.5), IQR: 5.3	39.2 (26.1–47.7), IQR: 3.9	< 0.001
INR	1.1 (0.9–1.5), IQR: 0.2	1.1 (0.1–1.5), IQR: 0.2	> 0.05
Creatinine mg/dL	0.8 (0.1–1.2), IQR:0.2	0.8 (0.1–1.3), IQR: 0.2	> 0.05
GFR mL/min	97 (47–161), IQR: 23	96 (41–125), IQR: 23	> 0.05
Leukocyte number/L	6.0 (2.2–11.5), IQR: 2.4	6.2 (1.9–12.8), IQR: 2.5	> 0.05
C-reactive protein mg/L	2.9 (2.9–29.9), IQR: 0.0	2.9 (2.9–20.4), IQR: 0.0	> 0.05
Procalcitonin ng/mL	0.07 (0.01–11.02), IQR: 0.08	0.03 (0.01–0.14), IQR: 0.05	< 0.001
Platelet number/nL	165 (38–364), IQR: 155	169 (38–391), IQR: 150	> 0.05
High-density lipoprotein mg/dL	53 (22–103)^53^, IQR: 19	52 (23–85)^57^, IQR: 18	> 0.05
Low-density lipoprotein mg/dL	85 (31–204)^53^, IQR: 56	116 (47–243)^54^. IQR: 61	< 0.001
Viral load	7 × 10^5^ (23–25 × 10^6^), IQR: 19 × 10^5^	1 (1–10), IQR: 0	< 0.001

**Fig. 7:**
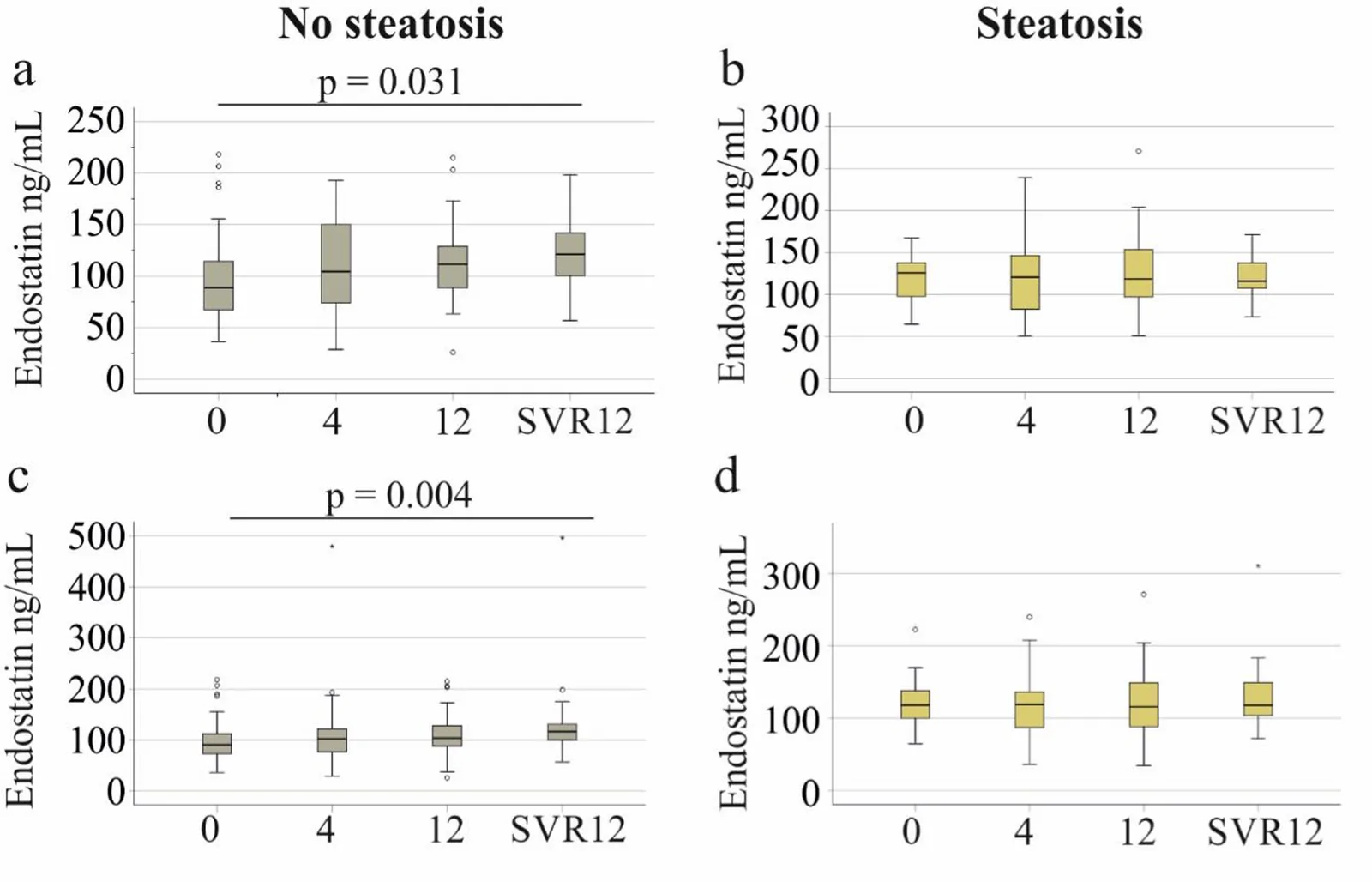
Serum endostatin levels during direct-acting antiviral therapy: (**a**) Endostatin serum levels before therapy start (0), at 4 weeks after therapy start (4), at therapy end (12), and at sustained virological response 12 (SVR12) in 30 patients without liver steatosis where serum was available at all time points; (**b**) Endostatin serum levels before therapy start (0), at 4 weeks after therapy start (4), at therapy end (12), and at SVR12 in 28 patients with liver steatosis where serum was available at all time points; (**c**) Endostatin serum levels of 48 patients before therapy, 53 at 4 weeks, 58 at 12 weeks after therapy start, and 48 at SVR12, all without liver steatosis; (**d**) Serum endostatin levels of 44 patients before therapy, 47 at 4 weeks, 48 at 12 weeks after therapy start, and 37 at SVR12, all with liver steatosis. Small circles and asterisks above the boxes mark outliers. Statistical test: Friedman test (a, c) and Kruskal–Wallis test (b, d)

In the 30 patients without steatosis, serum endostatin levels increased during therapy and were higher at SVR12 than before therapy (121 (57–198) ng/mL vs. 89 (36–218) ng/mL; Fig. 7a). The difference in endostatin levels between baseline (126 (65–168) ng/mL) and SVR12 (115 (73–171) ng/mL) was not significant in the subgroup of patients with fatty liver (28 patients; *p* = 0.685; Fig. 7b).

Including all patients with available serum, there were 48 patients before therapy, 53 at 4 weeks, 58 at 12 weeks after therapy start, and 48 at SVR12, all without liver steatosis. Serum endostatin was significantly higher at SVR12 than at pretreatment (Fig. 7c). In patients with steatosis, there were 44 patients before therapy, 47 at 4 weeks, 48 at 12 weeks after therapy start, and 37 at SVR12. Serum endostatin levels did not change over this period (*p* = 0.869; Fig. 7d).

### Serum endostatin levels at SVR12

**Fig. 8:**
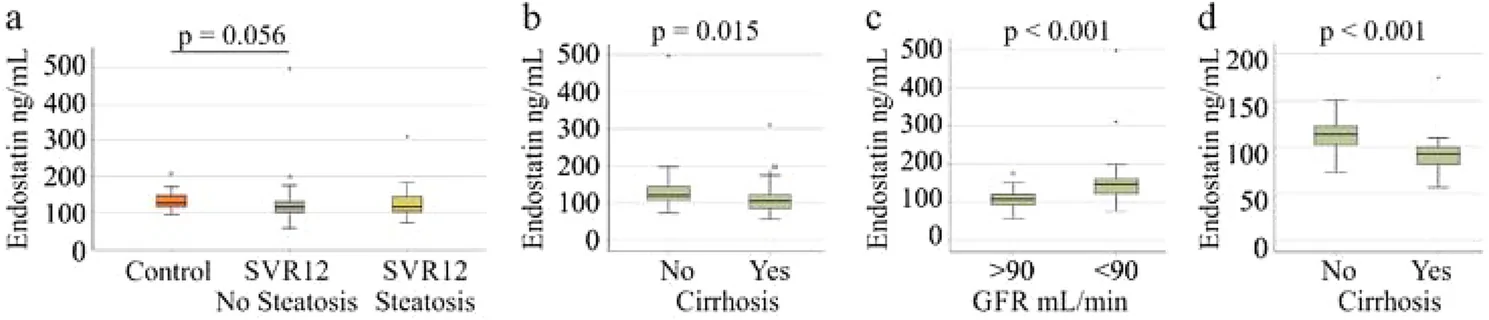
Serum endostatin levels at sustained virological response 12 (SVR12) according to steatosis and cirrhosis: (a) Serum endostatin levels of the 30 controls, 48 patients without liver steatosis, and 37 with liver steatosis at SVR12; (b) Serum endostatin levels of 31 patients with and 54 without liver cirrhosis defined by ultrasound at SVR12; (c) Serum endostatin levels of patients with cirrhosis with GFR > 90 mL/min (53 patients) and GFR < 90 mL/min (32 patients); (d) Serum endostatin levels in 17 patients with and 36 patients without liver cirrhosis in the subgroup with GFR > 90 mL/min. Small circles and asterisks above the boxes mark outliers. Statistical test: Kruskal–Wallis test (a) and Mann–Whitney U test (b-d).

At SVR12, serum endostatin levels in controls (129 (95–208) ng/mL), patients with steatosis (116 (72–310) ng/mL), and those without steatosis (115 (57–496) ng/mL) were similar (*p* = 0.058). Patients without steatosis tended to have lower serum endostatin levels at SVR12 than controls (*p* = 0.056; Fig. 8a).

At SVR12, serum endostatin levels in patients with liver cirrhosis (31 patients; 105 (57–310) ng/mL) were lower than in those without (54 patients; 121 (73–496) ng/mL; *p* = 0.024; Fig. 8b). This was not significant after excluding patients with liver steatosis, where 16 patients had cirrhosis (*p* = 0.050).

Patients with liver cirrhosis were older (*p* = 0.013). However, among patients older than 60 years, age (*p* = 0.093) and serum endostatin did not differ between patients with cirrhosis (15 patients) and those without cirrhosis (16 patients; *p* = 0.338).

At SVR12, patients with GFR > 90 mL/min (53 patients) had lower serum endostatin than those with GFR < 90 mL/min (32 patients; *p* < 0.001; Fig. 8c). Cirrhosis (17 patients) was related to lower serum endostatin in patients with GFR > 90 mL/min (*p* < 0.001; Fig. 8d).

At SVR12, sex, BMI, platelet count, leukocytes, GFR, steatosis (Yes/No), and cirrhosis (Yes/No) predicted serum endostatin levels in the entire cohort (F (7,62) = 18.224, *p* < 0.001). Sex (*p* < 0.001), BMI (*p* = 0.017), cirrhosis (*p* = 0.001), and GFR (*p* < 0.001) were significant. In the non-steatotic patients, sex, BMI, platelet count, leukocytes, GFR, steatosis, and cirrhosis also predicted endostatin (F(6,33) = 15.344, *p* < 0.001); sex (*p* = 0.046), cirrhosis (*p* = 0.009), and GFR (*p* < 0.001) were significant. This was also the case in steatotic patients (F(6,23) = 6.378, *p* < 0.001), where sex (*p* = 0.003) and GFR (*p* < 0.001) were significant.

Endostatin levels of males and females at SVR12 were similar (*p* = 0.433). Levels negatively correlated with GFR (*r* = -0.479, *p* < 0.001) but not with BMI (*r* = -0.045, *p* = 0.707).

### Serum endostatin levels at SVR12 in relation to DAA combinations

**Fig. 9:**
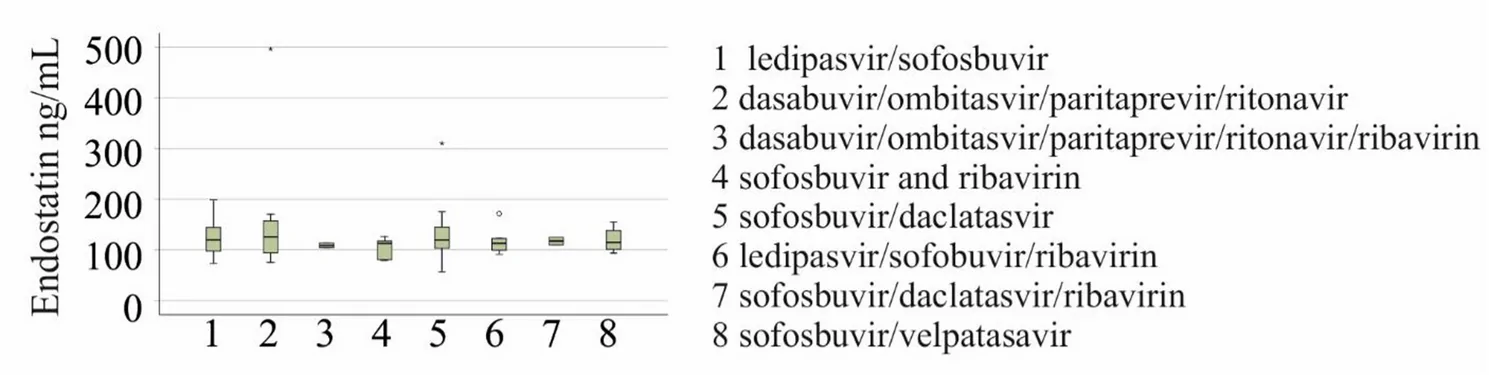
Serum endostatin levels at sustained virological response 12 (SVR12) according to drug combinations

Effects of the DAA drug combinations on serum endostatin levels at SVR12 were also analyzed. The 21 patients with ledipasvir/sofosbuvir, the 12 patients with dasabuvir/ombitasvir/paritaprevir/ritonavir, the 2 patients with dasabuvir/ombitasvir/paritaprevir/ritonavir/ribavirin, the 5 patients with sofosbuvir/ribavirin, the 6 patients with ledipasvir/sofobuvir/ribavirin, the 33 patients with sofosbuvir/daclatasvir, the 2 patients with sofosbuvir/daclatasvir/ribavirin, and the 4 patients with sofosbuvir/velpatasavir had similar serum endostatin levels at SVR12 (*p* = 0.963; Fig. 9). Steatosis prevalence was similar across drug combinations (*p* = 0.629).

## Discussion

This study shows that serum endostatin levels in patients with HCV and liver steatosis are comparable to those in healthy controls, whereas levels are reduced in HCV patients without fatty liver and normalize after viral eradication. In patients with HCV, serum endostatin levels do not vary with increasing fibrosis stage, as assessed non-invasively. However, they increase with renal impairment, which represents a significant confounding factor when interpreting serum endostatin levels.

Endostatin in circulation is thought to be derived primarily from the liver [16], suggesting that the reduced levels observed in HCV infection may result from diminished hepatic release. This reduction could stem from decreased collagen XVIII expression or impaired C-terminal proteolysis mediated by cathepsin L, cathepsin S, or matrix metalloproteinases [16]. Hepatocytes expressing HCV proteins show markedly reduced cathepsin S expression [39], which may contribute to diminished collagen XVIII proteolysis and, consequently, reduced hepatic release of endostatin into the circulation. HCV infection has also been shown to decrease cathepsin S levels in monocytes and dendritic cells [44, 45], suggesting that immune cells may further impair collagen XVIII processing. This hypothesis is supported by observations that serum endostatin levels are lower in patients with HCV infection and return to normal after viral eradication. However, direct experimental evidence confirming this mechanism is still lacking. Moreover, no direct evidence shows that the liver is the major organ controlling serum endostatin levels.

The pathways underlying the decline in endostatin levels during HCV infection may be altered in patients with concomitant hepatic steatosis. Indeed, cathepsin S expression has been reported to be increased in non-alcoholic fatty liver disease [46]. Whether this increase counteracts the HCV-induced suppression of cathepsin S remains to be determined. Furthermore, it is unclear why patients with steatosis at SVR12 do not exhibit higher endostatin levels than healthy controls, despite the presumed restoration of cathepsin S activity following viral clearance. However, collagen XVIII expression and the activity of the enzymes involved in its proteolytic processing have not been directly compared between HCV patients and healthy controls, nor have they been evaluated in HCV patients with versus without steatosis. One study showed a decline in collagen XVIII in the cirrhotic liver of patients with HCV, but did not compare it with healthy livers or differentiate between patients with and without steatosis [47].

Liver steatosis is common in HCV infection and may result from the virus itself and/or metabolic factors. Insulin resistance, arising from HCV infection and/or obesity, may further exacerbate hepatic lipid accumulation [48, 49]. In our cohort, the underlying cause of liver steatosis was not determined. We assessed hepatic steatosis using conventional B-mode ultrasonography, the only available method during the study period, which has limited sensitivity for mild steatosis despite good specificity and improved performance in higher-grade steatosis [40]. Furthermore, because metabolic dysfunction–associated steatotic liver disease (MASLD) was not systematically documented, we could not evaluate metabolic liver disease as a potential modifier of the observed associations. Notably, current clinical evidence directly linking serum endostatin to MASLD remains limited [16]. Endostatin has been described as protective against diet-induced obesity and obesity-related metabolic diseases such as insulin resistance and hepatic steatosis [7]. However, in our HCV patients, serum endostatin levels did not correlate with body mass index, a key risk factor for metabolic liver steatosis [50], suggesting that in chronic HCV, endostatin levels are not linked to obesity. Moreover, in patients with HCV, serum endostatin levels were comparable between patients with and without type 2 diabetes, suggesting that insulin resistance is also not a major confounding variable.

The effect of steatosis on the circulating proteome in patients with HCV is largely unknown. One study showed that HCV patients with liver steatosis had higher caspase activation in serum, which may serve as a non-invasive marker of steatosis [51]. Moreover, adiponectin levels are reduced in HCV patients with steatosis [52]. Endostatin has been reported to inhibit VEGF expression [5, 53], and in HCV-infected individuals with steatosis, serum VEGF concentrations were higher than in patients without steatosis. VEGF levels decreased at SVR12 in patients with and without liver steatosis, but remained higher in patients with steatosis [54].

Endostatin kinetics are similar to those of epidermal growth factor (EGF): they are higher in steatotic than in non-steatotic HCV patients and increase only in the latter group at SVR12, remaining unchanged in patients with steatosis [54]. However, the mechanisms maintaining normal endostatin levels in patients with steatosis, which otherwise decline in HCV infection, remain unclear.

Endostatin has been shown to block VEGF signaling and exert antifibrotic effects [16]. However, the link between liver steatosis and the progression of liver fibrosis remains controversial. Some studies indicate a positive association with advancing fibrosis and the onset of HCC, whereas others have found no significant relationship [55–57]. The German Hepatitis C Registry is a nationwide, multicenter, real-world cohort. This analysis included 8,789 individuals with HCV, stratified according to the presence of steatosis. Within this cohort, steatosis was not associated with a significantly higher rate of fibrosis progression in patients with HCV [57]. Previous studies have also reported an increased risk of fibrosis and HCC in HCV patients with liver steatosis [55, 56]. In our cohort, patients with and without steatosis did not differ in SWE or FIB-4 scores or in the prevalence of cirrhosis, consistent with Rau et al. [57].

Our study found no increase in serum endostatin levels in patients with advanced fibrosis, as diagnosed by SWE and the FIB-4 score, nor did they change in patients with ultrasound-diagnosed liver cirrhosis. Notably, in patients with normal GFR, serum endostatin levels were reduced in patients with cirrhosis. The assumption that the liver is a main source of serum endostatin levels is largely consistent with lower levels in cirrhosis, similar to the decline of C-reactive protein, a hepatocyte-produced protein, in cirrhosis [58].

However, other studies found higher serum endostatin levels to be a measure of liver fibrosis in patients with HCV. In Abdelgawad et al., serum endostatin levels in patients with HCV, measured before antiviral therapy, and advanced fibrosis were about 150% of those in patients with mild fibrosis [19]. Although liver biopsy, used in this study to stage liver fibrosis, provides a more direct assessment than the non-invasive methods used in our study, steatosis and renal function were not accounted for, despite their high prevalence among patients with hepatitis and the association of renal impairment with higher endostatin levels [22]. Another limitation is the lack of serum endostatin measurements in healthy controls [19].

Mohsen et al. showed that serum endostatin is about 130-fold higher in HCC (0.7 compared to 90 pg/ml) than in healthy controls. In chronic HCV-infected patients, endostatin levels were about 5-10-fold higher [18]. This paper did not report any details of the patient cohort [18], so we cannot compare it with our cohort. Other studies have reported comparable serum endostatin levels in patients with cirrhosis of various disease etiologies compared to patients with chronic liver disease and/or healthy controls [11, 13, 14]. Thus, our finding of unchanged serum endostatin levels in patients with cirrhosis aligns with several previous studies. Yegin et al. reported lower endostatin in cirrhosis patients than in controls; however, this poster abstract does not report disease etiology or patient characteristics [15]. In our cohort of HCV patients with normal GFR, serum endostatin levels were reduced in cirrhosis, but it is unclear whether all patients in the Yegin et al. study had normal renal function [15].

Higher endostatin in patients with renal impairment has been consistently reported [22]. In our study, endostatin levels were higher in HCV-infected patients with a GFR < 90 mL/min than in those with a GFR > 90 mL/min. This association was observed both before treatment and at SVR12 and was also evident among patients with HCV-related cirrhosis. However, in critically ill patients with cirrhosis, the association between renal impairment and higher endostatin levels appeared to be attenuated [17], suggesting that the combination of severe systemic illness and cirrhosis, rather than hepatic dysfunction alone, may underlie the observed effect on endostatin levels.

Non-invasive diagnosis of cirrhosis using the FIB-4 score has moderate diagnostic accuracy, and the FIB-4 score and SWE scores also seem to be affected by inflammation, as both decline after viral cure [27, 59]. Liver biopsy also has several limitations [60], and differences in the methods used to assess liver fibrosis stage may contribute to the inconsistent findings reported across studies. In the present study, liver fibrosis was assessed using the FIB-4 score and SWE, with consistent results observed for serum endostatin levels.

Linear regression showed that GFR and steatosis significantly predicted serum endostatin levels in our HCV cohort. GFR was similar between patients with and without steatosis and therefore did not explain the differences in endostatin levels. Notably, platelet number was positively correlated with serum endostatin levels but did not significantly predict it. Platelet count was comparable between patients with and without steatosis and therefore did not explain differences in serum endostatin levels.

Partial correlation analysis, adjusted for age—which showed a positive association with serum endostatin—revealed significant negative relationships between endostatin and the MELD score, bilirubin, INR, GFR, and HDL levels, and positive correlations with leukocyte and platelet count. Platelets store and release endostatin upon thrombin stimulation [61], which may explain the positive association of endostatin and platelet number. In patients with liver disease, platelet count and INR often show opposite associations with disease severity [62], consistent with the negative association between endostatin and INR. The MELD score is calculated from INR, bilirubin, and creatinine and is thus negatively associated with serum endostatin levels. Leukocyte count is positively correlated with platelet number (*r* = 0.650, *p* < 0.001), which may explain its association with endostatin levels. We have no clear explanation for the weak, unconfirmed correlation between endostatin and HDL. Because the MELD score, bilirubin, albumin, INR, HDL, platelet, and leukocyte counts were similar in patients with and without liver steatosis, these routine markers do not appear to account for the higher endostatin levels in steatotic HCV.

One study reported lower serum endostatin in splenectomized patients [23], while serum endostatin levels did not correlate directly with spleen size in our cohort. This suggests that age, liver steatosis, renal function, and possibly spleen function can affect serum endostatin levels, so these factors should be accounted for. Viral genotype, viral titer, and DAA combinations did not affect serum endostatin levels, so we excluded them as confounders.

At SVR12, sex, BMI, cirrhosis, and GFR predicted serum endostatin in the entire cohort, and further study has to evaluate the effect of sex and BMI on serum endostatin levels.

Differences between male and female patients were significant but small and require verification in larger cohorts. Moreover, age-adjusted comparisons included few patients and have to be considered preliminary. A limitation of this study is the lack of liver biopsy samples from the patients, preventing assessment of whether hepatic endostatin is regulated in those with HCV. Hepatic steatosis was evaluated using conventional B-mode ultrasonography. Although this technique has good specificity, its sensitivity is limited for detecting mild steatosis, and its diagnostic performance improves with increasing hepatic fat accumulation. Moreover, the study did not document whether patients had MASLD. Serum samples were collected soon after viral clearance, without extended follow-up. The study did not record laboratory measures or body mass index for the controls, which further limits the study. However, all controls reported being healthy, and all had normal body weight. Furthermore, because this research was conducted at a single center, the results’ generalizability to broader populations is limited.

## Conclusions

In conclusion, this study found that HCV infection reduces serum endostatin levels exclusively in patients without steatosis, with levels returning to normal by SVR12. Serum endostatin is not a marker for assessing the stage of liver fibrosis in HCV-infected individuals, either before or after viral clearance. Furthermore, its positive association with renal impairment is an important confounding factor to consider when interpreting serum endostatin levels.

## Supplementary information

Below is the link to the electronic supplementary material.

Supplementary Material 1

## Data Availability

The datasets used and/or analyzed during the current study are available from the corresponding author on reasonable request.

## Abbreviations

ALT: Alanine aminotransferase
AST: Aspartate aminotransferase
BMI: Body mass index
CRP: C-reactive protein
DAA: direct-acting antivirals
FIB-4: fibrosis-4
GFR: glomerular filtration rate
HCC: hepatocellular carcinoma
HCV: hepatitis C virus
HDL: High-density lipoprotein
INR: International normalized ratio
LDL: Low-density lipoprotein
MELD: Model for End-Stage Liver Disease
SVR12: sustained virological response at 12 weeks
SWE: shear-wave elastography (SWE)
VEGF: vascular endothelial growth factor

## References

[1] de Torres M, Poynard T. Risk factors for liver fibrosis progression in patients with chronic hepatitis C. Ann Hepatol. 2003;2(1):5–11. 15094700

[2] Li H, Huang MH, Jiang JD, Peng ZG. Hepatitis C: From inflammatory pathogenesis to anti-inflammatory/hepatoprotective therapy. World J Gastroenterol. 2018;24(47):5297–311. 10.3748/wjg.v24.i47.5297 PMC6305530 30598575

[3] Bocca C, Novo E, Miglietta A, Parola M. Angiogenesis and Fibrogenesis in Chronic Liver Diseases. Cell Mol Gastroenterol Hepatol. 2015;1(5):477–88. 10.1016/j.jcmgh.2015.06.011 PMC5301407 28210697

[4] Anakha J, Prasad YR, Pande AH. Endostatin in disease modulation: From cancer to beyond. Vascul Pharmacol. 2025;158:107459. 10.1016/j.vph.2024.107459 39708990

[5] Tsuji T, Kelly NJ, Takahashi S, Leme AS, Houghton AM, Shapiro SD. Macrophage elastase suppresses white adipose tissue expansion with cigarette smoking. Am J Respir Cell Mol Biol. 2014;51(6):822–9. 10.1165/rcmb.2014-0083OC PMC4291548 24914890

[6] Lee C, Kim MJ, Kumar A, Lee HW, Yang Y, Kim Y. Vascular endothelial growth factor signaling in health and disease: from molecular mechanisms to therapeutic perspectives. Signal Transduct Target Ther. 2025;10(1):170. 10.1038/s41392-025-02249-0 PMC12086256 40383803

[7] Wang H, Chen Y, Lu XA, Liu G, Fu Y, Luo Y. Endostatin Prevents Dietary-Induced Obesity by Inhibiting Adipogenesis and Angiogenesis. Diabetes. 2015;64(7):2442–56. 10.2337/db14-0528 25605807

[8] Weber F, Treeck O, Mester P, Buechler C. Expression and Function of BMP and Activin Membrane-Bound Inhibitor (BAMBI) in Chronic Liver Diseases and Hepatocellular Carcinoma. Int J Mol Sci. 2023;24:3473. 10.3390/ijms24043473 PMC9964332 36834884

[9] Chen J, Liu DG, Yang G, Kong LJ, Du YJ, Wang HY, Li FD, Pei FH, Song JT, Fan YJ, et al. Endostar, a novel human recombinant endostatin, attenuates liver fibrosis in CCl4-induced mice. Exp Biol Med (Maywood). 2014;239(8):998–1006. 10.1177/1535370214532595 24872431

[10] Ren H, Li Y, Chen Y, Wang L. Endostatin attenuates PDGF-BB- or TGF-beta1-induced HSCs activation via suppressing RhoA/ROCK1 signal pathways. Drug Des Devel Ther. 2019;13:285–90. 10.2147/DDDT.S191617 PMC6333321 30666090

[11] Joo YY, Jang JW, Lee SW, Yoo SH, Kwon JH, Nam SW, Bae SH, Choi JY, Yoon SK. Circulating pro- and anti-angiogenic factors in multi-stage liver disease and hepatocellular carcinoma progression. Sci Rep. 2019;9(1):9137. 10.1038/s41598-019-45537-w PMC6591389 31235729

[12] Poon RT, Ho JW, Tong CS, Lau C, Ng IO, Fan ST. Prognostic significance of serum vascular endothelial growth factor and endostatin in patients with hepatocellular carcinoma. Br J Surg. 2004;91(10):1354–60. 10.1002/bjs.4594 15376182

[13] Sharma BK, Srinivasan R, Kapil S, Singla B, Saini N, Chawla YK, Chakraborti A, Duseja A, Kalra N, Dhiman RK. Serum levels of angiogenic and anti-angiogenic factors: their prognostic relevance in locally advanced hepatocellular carcinoma. Mol Cell Biochem. 2013;383(1–2):103–12. 10.1007/s11010-013-1759-7 23912396

[14] Uematsu S, Higashi T, Nouso K, Kariyama K, Nakamura S, Suzuki M, Nakatsukasa H, Kobayashi Y, Hanafusa T, Tsuji T, et al. Altered expression of vascular endothelial growth factor, fibroblast growth factor-2 and endostatin in patients with hepatocellular carcinoma. J Gastroenterol Hepatol. 2005;20(4):583–8. 10.1111/j.1440-1746.2005.03726.x 15836707

[15] Yegin E, Syikymbayev A, Ozdogan OC. Serum Endostatin Level Is Associated with Poor Survival in Patients with Advanced Stages of Hepatocellular Carcinoma. J Hepatol. 2013;58:S270–270.

[16] Grewal T, Buechler C. Anti-Angiogenic Features of Endostatin in Obesity, Liver Fibrosis, and Hepatocellular Carcinoma. Biomedicines 2026, 14(3):734 . 10.3390/biomedicines14030734 PMC13023974 41898378

[17] Fröhlich L, Fromm C, Läber L, Gepperth V, Schmid S, Mester MM, Pavel P, Buechler V. C: Liver Cirrhosis and Septic Shock Attenuate the Increase in Plasma Endostatin Concentrations That Is Associated with Acute Kidney Injury Requiring Dialysis *Biomedicines* 2026, 14(10): 2142 . 10.3390/biomedicines14102142 PMC13701869 42939948

[18] Mohsen MAA, Hussein NA, Ghazal AA, El-Ghandour MK, Farouk M, El-Wahab AEA, Yousef AI. Angiogenic output in viral hepatitis, C and B, and HCV-associated hepatocellular carcinoma. Alexandria J Med. 2014;50:235–40.

[19] Abdelgawad ME, El-Zakzok H, Salah M, El-Mezayen HA. Diagnostic accuracy of hepatocyte growth factor, Fas/CD95 and Endostatin for non-invasive assessment of hepatic fibrosis in biopsy-proven hepatitis C virus patients. Infect Genet Evol. 2020;85:104526. 10.1016/j.meegid.2020.104526 32890764

[20] Vukobrat-Bijedic Z, Husic-Selimovic A, Mehinovic L, Junuzovic D, Bijedic N, Sofic A, Bjelogrlic I, Mehmedovic A. Estimated Glomerular Filtration Rate (eGFR) Values as Predictor of Renal Insufficiency in Advanced Stages of Liver Diseases with Different Etiology. Med Arch. 2014;68(3):159–62. 10.5455/medarh.2014.68.159-162 PMC4240337 25568524

[21] Gluhovschi C, Velciov S, Buzas R, Petrica L, Bozdog G, Gadalean F, Gluhovschi A, Balgradean C, Vernic C, Sporea I. Is There any Difference Between the Glomerular Filtration Rate of Patients With Chronic Hepatitis B and C and Patients With Cirrhosis? Hepat monthly. 2013;13(4):e6789. 10.5812/hepatmon.6789 PMC3693541 23805157

[22] Li M, Popovic Z, Chu C, Kramer BK, Hocher B. Endostatin in Renal and Cardiovascular Diseases. Kidney Dis (Basel). 2021;7(6):468–81. 10.1159/000518221 PMC8613550 34901193

[23] Zuo T, Xie Q, Liu J, Yang J, Shi J, Kong D, Wang Y, Zhang Z, Gao H, Zeng DB, et al. Macrophage-Derived Cathepsin S Remodels the Extracellular Matrix to Promote Liver Fibrogenesis. Gastroenterology. 2023;165(3):746–61. e716. 10.1053/j.gastro.2023.05.039 37263311

[24] Vallet-Pichard A, Mallet V, Nalpas B, Verkarre V, Nalpas A, Dhalluin-Venier V, Fontaine H, Pol S. FIB-4: an inexpensive and accurate marker of fibrosis in HCV infection. Comparison with liver biopsy and fibrotest. Hepatology. 2007;46(1):32–6. 10.1002/hep.21669 17567829

[25] Vidili G, Arru M, Meloni P, Solinas G, Atzori S, Maida I. Comparison of 2D Shear Wave Elastography and Transient Elastography in Non-Invasive Evaluation of Liver Fibrosis in Hepatitis C Virus-Related Chronic Liver Disease. J Clin Med. 2024;13(14):4061. 10.3390/jcm13144061 PMC11278231 39064101

[26] Huynh T, Zhang J, Hu KQ. Hepatitis C Virus Clearance by Direct-acting Antiviral Results in Rapid Resolution of Hepatocytic Injury as Indicated by Both Alanine Aminotransferase and Aspartate Aminotransferase Normalization. J Clin Transl Hepatol. 2018;6(3):258–63. 10.14218/JCTH.2018.00014 PMC6160300 30271737

[27] Peschel G, Grimm J, Buechler C, Gunckel M, Pollinger K, Aschenbrenner E, Kammerer S, Jung EM, Haimerl M, Werner J, et al. Liver stiffness assessed by shear-wave elastography declines in parallel with immunoregulatory proteins in patients with chronic HCV infection during DAA therapy. Clin Hemorheology Microcirculation. 2021;79(4):541–55. 10.3233/CH-211193 34120896

[28] Thallapureddy K, Twitchell D, Ott K, Pedicone LD, Owo C, Kumar N, Gelfond J, Shankar N, Goros M, Kwok D, et al. The accuracy of FibroScan, FIB-4, and nonalcoholic fatty liver disease fibrosis score in predicting biopsy-defined fibrosis and steatosis across all fibrosis stages in patients with metabolic dysfunction associated steatotic liver disease. Med (Baltim). 2025;104(17):e42016. 10.1097/MD.0000000000042016 PMC12040034 40295262

[29] Moini M, Onofrio F, Hansen BE, Adeyi O, Khalili K, Patel K. Combination of FIB-4 with ultrasound surface nodularity or elastography as predictors of histologic advanced liver fibrosis in chronic liver disease. Sci Rep. 2021;11(1):19275. 10.1038/s41598-021-98776-1 PMC8481285 34588540

[30] Zeng H, Li L, Hou Z, Zhang Y, Tang Z, Liu S. Direct-acting Antiviral in the Treatment of Chronic Hepatitis C: Bonuses and Challenges. Int J Med Sci. 2020;17(7):892–902. 10.7150/ijms.43079 PMC7163356 32308542

[31] Verna EC, Morelli G, Terrault NA, Lok AS, Lim JK, Di Bisceglie AM, Zeuzem S, Landis CS, Kwo P, Hassan M, et al. DAA therapy and long-term hepatic function in advanced/decompensated cirrhosis: Real-world experience from HCV-TARGET cohort. J Hepatol. 2020;73(3):540–8. 10.1016/j.jhep.2020.03.031 32243960

[32] Sohn W, Park SY, Lee TH, Chon YE, Kim IH, Lee BS, Yoon KT, Jang JY, Lee YR, Yu SJ, et al. Effect of direct-acting antivirals on disease burden of hepatitis C virus infection in South Korea in 2007–2021: a nationwide, multicentre, retrospective cohort study. EClinicalMedicine. 2024;73:102671. 10.1016/j.eclinm.2024.102671 PMC11176940 38881570

[33] McPherson S, Hardy T, Dufour JF, Petta S, Romero-Gomez M, Allison M, Oliveira CP, Francque S, Van Gaal L, Schattenberg JM, et al. Age as a Confounding Factor for the Accurate Non-Invasive Diagnosis of Advanced NAFLD Fibrosis. Am J Gastroenterol. 2017;112(5):740–51. 10.1038/ajg.2016.453 PMC5418560 27725647

[34] Colombo S, Buonocore M, Del Poggio A, Jamoletti C, Elia S, Mattiello M, Zabbialini D, Del Poggio P. Head-to-head comparison of transient elastography (TE), real-time tissue elastography (RTE), and acoustic radiation force impulse (ARFI) imaging in the diagnosis of liver fibrosis. J Gastroenterol. 2012;47(4):461–9. 10.1007/s00535-011-0509-4 22223175

[35] Szakacs Z, Eross B, Soos A, Matrai P, Szabo I, Petervari E, Bajor J, Farkas N, Hegyi P, Illes A, et al. Baveno Criteria Safely Identify Patients With Compensated Advanced Chronic Liver Disease Who Can Avoid Variceal Screening Endoscopy: A Diagnostic Test Accuracy Meta-Analysis. Front Physiol. 2019;10:1028. 10.3389/fphys.2019.01028 PMC6711320 31481896

[36] Parikh ND, Mehta M, Tapper EB. FIB-4 and APRI for cirrhosis detection in a privately insured national cohort. JHEP Rep. 2024;6(1):100925. 10.1016/j.jhepr.2023.100925 PMC10701128 38074510

[37] Tapper EB, Parikh ND. Diagnosis and Management of Cirrhosis and Its Complications: A Review. JAMA. 2023;329(18):1589–602. 10.1001/jama.2023.5997 PMC10843851 37159031

[38] Indre MG, Leucuta DC, Lupsor-Platon M, Turco L, Ferri S, Hashim A, Orasan OH, Procopet B, Stefanescu H, Morelli MC, et al. Diagnostic accuracy of 2D-SWE ultrasound for liver fibrosis assessment in MASLD: A multilevel random effects model meta-analysis. Hepatology. 2025;82(2):454–69. 10.1097/HEP.0000000000001190 PMC12266795 39689354

[39] Ibacahe C, Correa-Burrows P, Burrows R, Barrera G, Kim E, Hirsch S, Jofre B, Blanco E, Gahagan S, Bunout D. Accuracy of a Semi-Quantitative Ultrasound Method to Determine Liver Fat Infiltration in Early Adulthood. Diagnostics (Basel) 2020, 10(6):431 . 10.3390/diagnostics10060431 PMC7345476 32630407

[40] Palmentieri B, de Sio I, La Mura V, Masarone M, Vecchione R, Bruno S, Torella R, Persico M. The role of bright liver echo pattern on ultrasound B-mode examination in the diagnosis of liver steatosis. Dig liver disease: official J Italian Soc Gastroenterol Italian Association Study Liver. 2006;38(7):485–9. 10.1016/j.dld.2006.03.021 16716779

[41] Shah I, Malik MO, Khan MJ, Fatima S, Baxendale RH, Habib SH. Endostatin Concentration In Plasma Of Healthy Human Volunteers. J Ayub Med Coll Abbottabad. 2017;29(2):200–6. 28718231

[42] Larsson A, Wegmann B, Ruge T, Alfredsson J, Östgren CJ, Lindahl TL. Elevated endostatin is associated with hypertension treatment, elevated high sensitivity CRP, increased waisthip ratio, and attenuated kidney function, but not with age, in a middleaged population. Global Cardiol 2025;)3(1):25–32.

[43] Peschel G, Grimm J, Gulow K, Muller M, Buechler C, Weigand K. Chemerin Is a Valuable Biomarker in Patients with HCV Infection and Correlates with Liver Injury. Diagnostics (Basel). 2020;10(11):974. 10.3390/diagnostics10110974 PMC7699464 33228201

[44] Kim H, Mazumdar B, Bose SK, Meyer K, Di Bisceglie AM, Hoft DF, Ray R. Hepatitis C virus-mediated inhibition of cathepsin S increases invariant-chain expression on hepatocyte surface. J Virol. 2012;86(18):9919–28. 10.1128/JVI.00388-12 PMC3446550 22761382

[45] Granato M, Lacconi V, Peddis M, Di Renzo L, Valia S, Rivanera D, Antonelli G, Frati L, Faggioni A, Cirone M. Hepatitis C virus present in the sera of infected patients interferes with the autophagic process of monocytes impairing their in-vitro differentiation into dendritic cells. Biochim Biophys Acta. 2014;1843(7):1348–55. 10.1016/j.bbamcr.2014.04.003 24726834

[46] van Mourik H, Li M, Baumgartner S, Theys J, Shiri-Sverdlov R. All Roads Lead to Cathepsins: The Role of Cathepsins in Non-Alcoholic Steatohepatitis-Induced Hepatocellular Carcinoma. Biomedicines 2022, 10(10):2351. 10.3390/biomedicines10102351 PMC9598942 36289617

[47] Baiocchini A, Montaldo C, Conigliaro A, Grimaldi A, Correani V, Mura F, Ciccosanti F, Rotiroti N, Brenna A, Montalbano M, et al. Extracellular Matrix Molecular Remodeling in Human Liver Fibrosis Evolution. PLoS ONE. 2016;11(3):e0151736. 10.1371/journal.pone.0151736 PMC4801190 26998606

[48] Roeb E. Non-alcoholic fatty liver diseases: current challenges and future directions. Ann Transl Med. 2021;9(8):726. 10.21037/atm-20-3760 PMC8106107 33987424

[49] Roingeard P. Hepatitis C virus diversity and hepatic steatosis. J Viral Hepatitis. 2013;20(2):77–84. 10.1111/jvh.12035 23301542

[50] Younossi Z, Tacke F, Arrese M, Chander Sharma B, Mostafa I, Bugianesi E, Wai-Sun Wong V, Yilmaz Y, George J, Fan J, et al. Global Perspectives on Nonalcoholic Fatty Liver Disease and Nonalcoholic Steatohepatitis. Hepatology. 2019;69(6):2672–82. 10.1002/hep.30251 30179269

[51] Seidel N, Volkmann X, Langer F, Flemming P, Manns MP, Schulze-Osthoff K, Bantel H. The extent of liver steatosis in chronic hepatitis C virus infection is mirrored by caspase activity in serum. Hepatology. 2005;42(1):113–20. 10.1002/hep.20747 15920717

[52] Durante-Mangoni E, Zampino R, Marrone A, Tripodi MF, Rinaldi L, Restivo L, Cioffi M, Ruggiero G, Adinolfi LE. Hepatic steatosis and insulin resistance are associated with serum imbalance of adiponectin/tumour necrosis factor-alpha in chronic hepatitis C patients. Aliment Pharmacol Ther. 2006;24(9):1349–57. 10.1111/j.1365-2036.2006.03114.x 17059516

[53] Wang X, Xu X, Tang L, Song S, Fan C. Interactions between Endostatin and Vascular Endothelial Growth Factor (VEGF) and Inhibition of Choroidal Neovascularization. Int J Mol Sci. 2007;8(1):61–9.

[54] Matotek RL, Zidovec-Lepej S, Salek N, Vince A, Papic N. The Impact of Liver Steatosis on Interleukin and Growth Factors Kinetics during Chronic Hepatitis C Treatment. J Clin Med 2024, 13(16):4849 . 10.3390/jcm13164849 PMC11355301 39200991

[55] Siphepho PY, Liu YT, Shabangu CS, Huang JF, Huang CF, Yeh ML, Yu ML, Wang SC. The Impact of Steatosis on Chronic Hepatitis C Progression and Response to Antiviral Treatments. Biomedicines 2021, 9(10):1491. 10.3390/biomedicines9101491 PMC8533513 34680608

[56] Asselah T, Rubbia-Brandt L, Marcellin P, Negro F. Steatosis in chronic hepatitis C: why does it really matter? Gut. 2006;55(1):123–30. 10.1136/gut.2005.069757 PMC1856395 16344578

[57] Rau M, Buggisch P, Mauss S, Boeker KHW, Klinker H, Muller T, Stoehr A, Schattenberg JM, Geier A. Prognostic impact of steatosis in the clinical course of chronic HCV infection-Results from the German Hepatitis C-Registry. PLoS ONE. 2022;17(6):e0264741. 10.1371/journal.pone.0264741 PMC9203066 35709466

[58] Pieri G, Agarwal B, Burroughs AK. C-reactive protein and bacterial infection in cirrhosis. Ann Gastroenterol. 2014;27(2):113–20. PMC3982625 24733601

[59] Leustean A, Popescu C, Nichita L, Tiliscan C, Arama V. Dynamics of APRI and FIB-4 in HCV cirrhotic patients who achieved SVR after DAA therapy. Exp Ther Med. 2021;21(1):99. 10.3892/etm.2020.9531 PMC7725022 33363610

[60] Joseph J. Serum Marker Panels for Predicting Liver Fibrosis - An Update. Clin Biochem Rev. 2020;41(2):67–73. 10.33176/AACB-20-00002 PMC7255312 32518428

[61] Ma L, Elliott SN, Cirino G, Buret A, Ignarro LJ, Wallace JL. Platelets modulate gastric ulcer healing: role of endostatin and vascular endothelial growth factor release. Proc Natl Acad Sci U S A. 2001;98(11):6470–5. 10.1073/pnas.111150798 PMC33492 11353854

[62] Kurisu K, Sato K, Matsuoka M, Otani M, Yoshiuchi K. Thrombocytopenia and PT-INR in patients with anorexia nervosa and severe liver dysfunction. Biopsychosoc Med. 2023;17(1):9. 10.1186/s13030-023-00269-2 PMC9996900 36890598

